# Pyrosequencing-Based Assessment of Bacterial Community Structure Along Different Management Types in German Forest and Grassland Soils

**DOI:** 10.1371/journal.pone.0017000

**Published:** 2011-02-16

**Authors:** Heiko Nacke, Andrea Thürmer, Antje Wollherr, Christiane Will, Ladislav Hodac, Nadine Herold, Ingo Schöning, Marion Schrumpf, Rolf Daniel

**Affiliations:** 1 Department of Genomic and Applied Microbiology, Institute of Microbiology and Genetics, Georg-August University Göttingen, Göttingen, Germany; 2 Göttingen Genomics Laboratory, Institute of Microbiology and Genetics, Georg-August University Göttingen, Göttingen, Germany; 3 Experimental Phycology and Culture Collection of Algae at the University of Göttingen, Göttingen, Germany; 4 Max Planck Institute for Biogeochemistry, Jena, Germany; 5 Institute of Ecology, Friedrich-Schiller-University, Jena, Germany; Argonne National Laboratory, United States of America

## Abstract

**Background:**

Soil bacteria are important drivers for nearly all biogeochemical cycles in terrestrial ecosystems and participate in most nutrient transformations in soil. In contrast to the importance of soil bacteria for ecosystem functioning, we understand little how different management types affect the soil bacterial community composition.

**Methodology/Principal Findings:**

We used pyrosequencing-based analysis of the V2-V3 16S rRNA gene region to identify changes in bacterial diversity and community structure in nine forest and nine grassland soils from the Schwäbische Alb that covered six different management types. The dataset comprised 598,962 sequences that were affiliated to the domain Bacteria. The number of classified sequences per sample ranged from 23,515 to 39,259. Bacterial diversity was more phylum rich in grassland soils than in forest soils. The dominant taxonomic groups across all samples (>1% of all sequences) were *Acidobacteria*, *Alphaproteobacteria*, *Actinobacteria*, *Betaproteobacteria*, *Deltaproteobacteria*, *Gammaproteobacteria*, and *Firmicutes*. Significant variations in relative abundances of bacterial phyla and proteobacterial classes, including *Actinobacteria*, *Firmicutes*, *Verrucomicrobia*, *Cyanobacteria*, *Gemmatimonadetes* and *Alphaproteobacteria,* between the land use types forest and grassland were observed. At the genus level, significant differences were also recorded for the dominant genera *Phenylobacter*, *Bacillus*, *Kribbella*, *Streptomyces*, *Agromyces*, and *Defluviicoccus*. In addition, soil bacterial community structure showed significant differences between beech and spruce forest soils. The relative abundances of bacterial groups at different taxonomic levels correlated with soil pH, but little or no relationships to management type and other soil properties were found.

**Conclusions/Significance:**

Soil bacterial community composition and diversity of the six analyzed management types showed significant differences between the land use types grassland and forest. Furthermore, bacterial community structure was largely driven by tree species and soil pH.

## Introduction

Soils are considered to be the most diverse microbial habitat on Earth with respect to species diversity and community size. Bacteria are the most abundant group of microorganisms in soil [1]. The calculated number of distinct bacterial genomes ranges from 2,000 to 18,000 per gram of soil [2]. Although the importance of bacteria for ecosystem functions and maintaining soil quality in agriculturally managed systems has long been recognized, the influence of land use type and management type on soil bacterial communities is poorly understood. In a recent pyrosequencing survey, bacterial diversity of forest soil was more phylum rich compared to agricultural soils, which were more species rich [3]. Furthermore, it has been described that *Bacteroidetes* were more predominant in Pullman soil in agricultural systems than in the same soil under non-disturbed conditions, whereas the opposite trend was found for *Actinobacteria* [4]. It has been reported that land use indirectly affects the bacterial community structure by modification of soil properties [5]. Other studies also indicated that soil properties are important drivers of soil bacterial community structure [6], but soil pH appears to be a major factor influencing community composition [7]. This influence of soil pH has been recognized at coarse levels of taxonomic resolution [8], but also within individual phyla [9]. In addition, it has been shown that the type of plant species [10], soil type [11], soil texture [12], and nitrogen availability [13] can affect bacterial community structure. Tree species influences on soil bacterial communities are indicated by previous studies [14], but detailed information on the affected bacterial groups and degree of these influences is still lacking.

In most previous studies the effects of land use and soil properties on soil bacterial communities have been assessed by employing traditional molecular methods such as Sanger sequencing-based analysis of 16S rRNA gene libraries or fingerprinting methods [15]. These approaches are often limited to the analysis of a relatively small number of clones and a few different soil samples. Taking into account the large bacterial community size and the heterogeneity of soils, only a tiny fraction of the bacterial diversity was unraveled by these studies. Recently, high-throughput pyrosequencing of 16S rRNA gene fragments has been applied for in-depth analysis of soil bacterial communities [3], [4]. However, most of the available pyrosequencing studies do not allow a statistical assessment of land use and management effects on soil bacterial communities, as analyses of replicates were often not performed.

In this report, we applied pyrosequencing of the V2-V3 16S rRNA gene region to analyze bacterial community structure in A horizons of forest and grassland sites, which varied in management type. A horizons are mineral soil horizons formed at the surface or below an O horizon, which is dominated by organic material consisting of undecomposed or partially decomposed litter. A horizons are often characterized by accumulation of humidified organic matter [16]. It has been shown that analysis of the V2-V3 region provides a taxonomic resolution ranging from the phylum level to the genus level [17]. Thus, it is possible to detect variations in bacterial communities at different taxonomic levels. We analyzed 18 different soil samples derived from the Schwäbische Alb, which is one of the three German Biodiversity Exploratories [18]. Schwäbische Alb is a mosaic of forest and grasslands with a higher proportion of grassland. This is due to traditional sheep herding. We determined soil bacterial community structure in A horizons of 9 forest and 9 grassland sites. The selected grassland and forest sites covered a range of 6 different management types. Triplicates of the different management types were analyzed, which is an important feature of this study, as it allows statistical analysis of management effects on soil bacterial communities. For each sample, the relative abundance and the distribution of bacterial groups were determined. Subsequently, we correlated variations in the relative abundances with land use type, management type, and soil properties.

## Results and Discussion

### General characteristics of the soil samples

In this study, we assessed and compared the composition of soil bacterial communities present in the A horizons of 18 soil samples derived from forest and grassland sites of the Schwäbische Alb (Germany) by large-scale pyrosequencing-based analysis of 16S rRNA gene sequences. The soil samples represented triplicates of 6 different management types, which encompassed spruce age class forest (SAF1-3), beech age class forest (BAF1-3), unmanaged beech forest (BF1-3), fertilized intensely managed grassland (FUG1-3), fertilized mown pasture grazed by horse and cattle (FMG1-3), and unfertilized pasture grazed by sheep (UPG1-3) (Tables 1 and S1). The soil groups of the forest soils and the grassland soils were Cambisols and Leptosols, respectively (Table 1). In addition, soil properties such as total nitrogen (N) content, organic carbon (OC) content, pH, and soil texture were determined. The soils had overall low sand (71±64 g kg^−1^) and highly variable clay contents with values ranging from 188 to 670 g kg^−1^ (average 412 g kg^−1^). Similarly, OC contents showed a huge variability (68±16 g kg^−1^). Total N contents were on average lower in forest sites than in grassland sites and C/N ratios were accordingly higher (14±1 forest and 11±1 grassland) (Table 1). The forest samples showed lower pH values than the grassland soils, which were all, except FMG1, near neutral. The analysis of differences of soil properties and management types by employing one-way analysis of variance and Tukey pair-wise comparisons showed that the analyzed management types did not vary significantly in OC, total N, and soil texture (Table S2). The only significant difference between management types was observed for the pH values, which were higher in unfertilized pastures grazed by sheep (6.9±0.4) than in spruce age class forests (4.7±0.9).

**Table 1:** Physical and geochemical characteristics of the analyzed grassland and forest soil samples.

Management type	Sample	Soil group	pH	OC (g kg^−1^)	Total N (g kg^−1^)	C:N ratio	Gravimetric water content (%)	Particle size (g kg^−1^)		
								Sand	Silt	Clay
Spruce age class forest	SAF1	Cambisol	3.30	64.57	3.97	16.26	62.8	26	668	306
Spruce age class forest	SAF2	Cambisol	4.55	65.19	4.35	14.99	65.2	43	446	511
Spruce age class forest	SAF3	Cambisol	5.04	74.68	5.14	14.53	76.5	60	445	495
Beech age class forest	BAF1	Cambisol	6.38	78.50	6.01	13.06	75.1	70	534	396
Beech age class forest	BAF2	Cambisol	4.52	57.53	4.45	12.93	70.4	47	587	368
Beech age class forest	BAF3	Cambisol	5.36	39.05	3.15	12.40	50.8	107	575	318
Unmanaged beech forest	BF1	Cambisol	4.87	77.62	5.54	14.01	75.7	109	371	520
Unmanaged beech forest	BF2	Cambisol	5.10	105.00	6.77	15.51	96.6	34	296	670
Unmanaged beech forest	BF3	Cambisol	6.37	60.03	4.49	13.37	54.9	56	495	449
Fertilized intensely managed grassland	FUG1	Leptosol	6.71	77.09	7.58	10.17	66.2	38	543	419
Fertilized intensely managed grassland	FUG2	Leptosol	6.92	72.25	7.18	10.06	59.6	139	646	215
Fertilized intensely managed grassland	FUG3	Leptosol	6.32	53.74	5.18	10.37	57.2	25	449	526
Fertilized mown pasture, horse and cattle	FMG1	Leptosol	5.11	51.61	5.35	9.65	57.5	80	475	445
Fertilized mown pasture, horse and cattle	FMG2	Leptosol	6.36	85.16	7.87	10.82	76.4	56	694	250
Fertilized mown pasture, horse and cattle	FMG3	Leptosol	6.14	68.17	6.67	10.22	64.0	32	492	476
Unfertilized pasture, sheep	UPG1	Leptosol	7.24	40.85	3.65	11.19	46.7	282	530	188
Unfertilized pasture, sheep	UPG2	Leptosol	6.45	81.15	7.41	10.95	74.3	18	384	598
Unfertilized pasture, sheep	UPG3	Leptosol	6.65	68.89	5.82	11.84	67.6	44	684	272

### General analyses of the pyrosequencing-derived dataset

Profiling of pylogenetic diversity and community composition by large-scale pyrosequencing of 16S rRNA gene sequences provides more sequence information compared to traditional Sanger sequencing of 16S rRNA gene clone libraries [19]. Although the per-base error rate of pyrosequencing of 16S rRNA genes is not higher than that of Sanger sequencing, the intrinsic error rate of pyrosequencing might lead to overestimation of the number of rare phylotypes. Since each pyrosequencing read is treated as an unique identifier of a community member and correction by assembly and sequencing depth applied during genome projects is not feasible, errors can result in overestimation of diversity [20], [21]. To minimize the overestimation of rare phylotypes, we used quality filtering of the pyrosequencing-derived dataset, and clustering and diversity estimates were performed at genetic divergences of ≥3% [21]. Alpha diversity analysis was performed at the same level of surveying effort (22,000 sequences per sample). In addition, denoising of each sequence subset was performed to avoid overestimation of operational taxonomic units (OTUs) and diversity [22], [23]. The pyrosequencing-based analysis of the V2-V3 region of the 16S rRNA genes resulted in recovery of 599,284 high quality sequences with a read length of ≥200 bp across all 18 samples. The average read length was 255 bp. The number of sequences per sample ranged from 23,519 to 39,273 with an average of 33,275 (Table S1). We were able to assign 598,962 sequences to the domain Bacteria and to classify 474,868 (79.3%) of these sequences below the domain level. Taking into account the number of sequences per sample and the number of analyzed sequences, the size of this study exceeded other published studies on pyrosequencing-based determination of soil bacterial community composition [3], [4], [7].

### Bacterial diversity and richness

**Figure 1:**
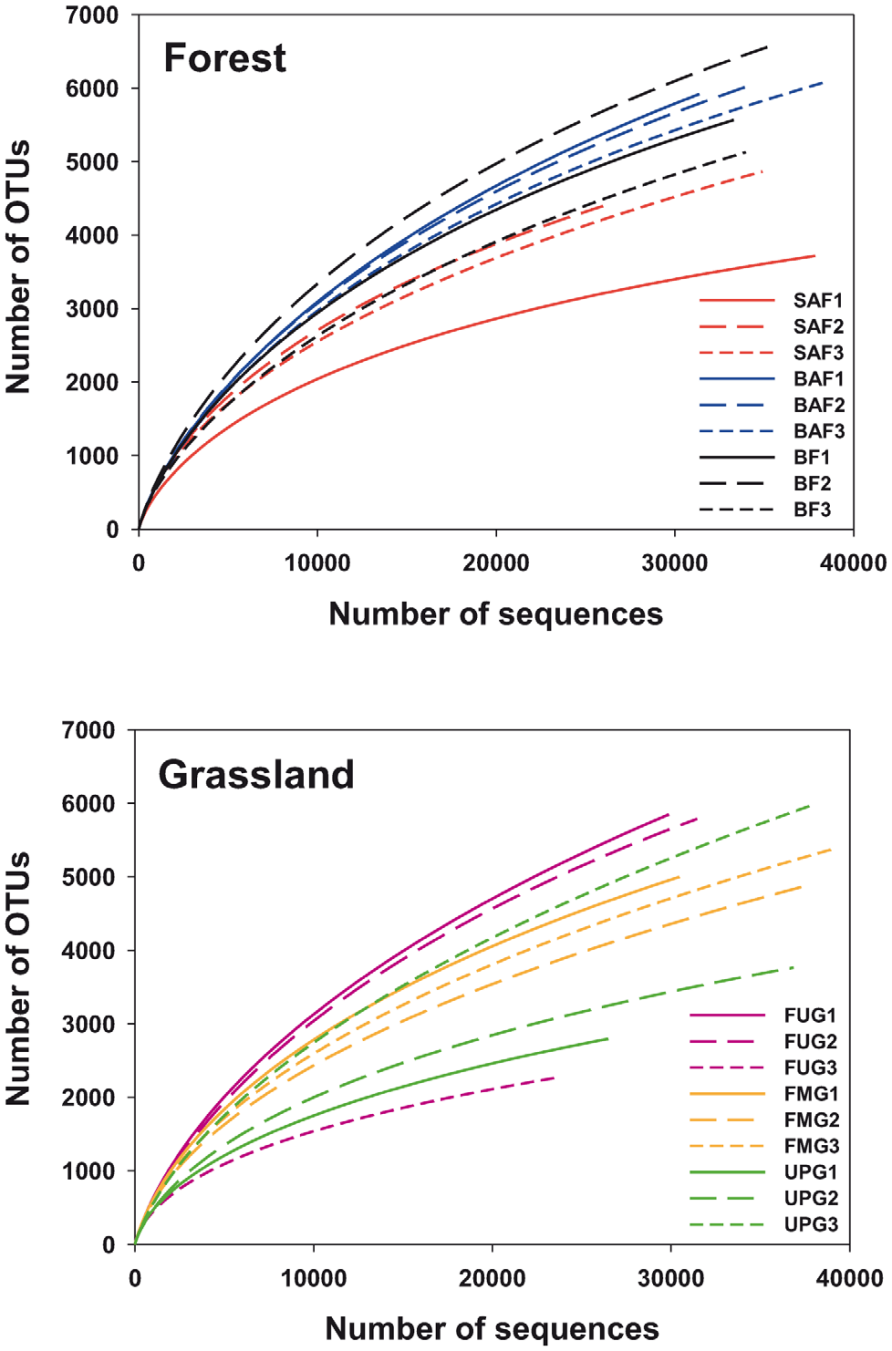
Rarefaction curves indicating the observed number of operational taxonomic units (OTUs) at a genetic distance of 3% in different forest and grassland soils. The spruce age class forest (SAF1-3), beech age class forest (BAF1-3), and unmanaged beech forest (BF1-3) sampling sites are marked by the red, blue, and black color, respectively. The fertilized intensely managed grassland (FUG1-3), fertilized mown pasture grazed by horse and cattle (FMG1-3), and unfertilized pasture grazed by sheep (UPG1-3) sampling sites are shown in purple, orange, and green, respectively.

**Figure 2:**
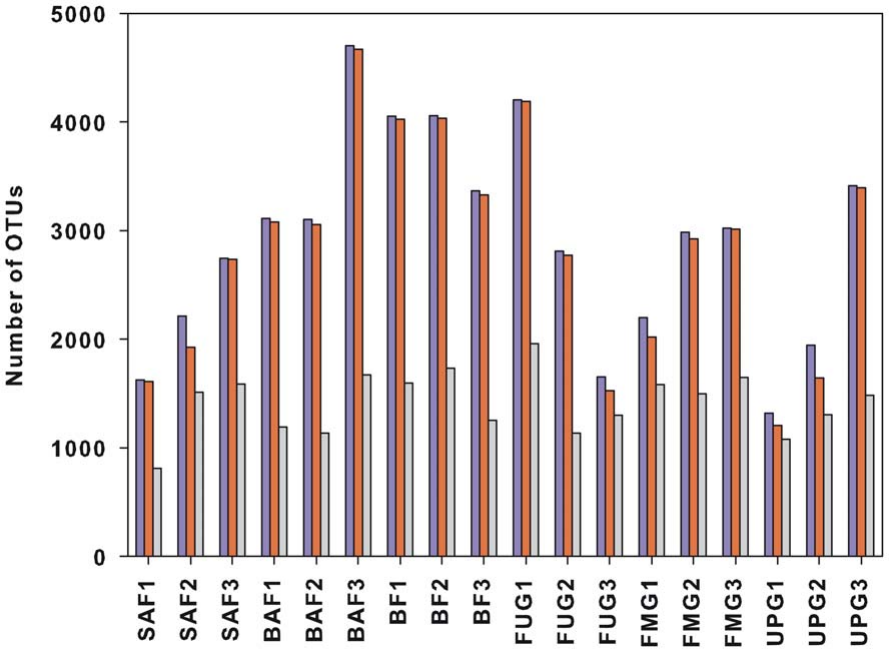
Bacterial richness estimates of German grassland and forest soils representing different management types at a genetic distance of 3%. Richness is expressed as number of observed unique OTUs. In addition, richness has been estimated by the abundance-based coverage estimator (ACE), which is a nonparametric richness estimator based on distribution of abundant (>10) and rare (≤10) OTUs, and the richness estimator Chao1, which is a nonparametric richness estimator based on distribution of singletons and doubletons. Richness prediction from Chao1 is colored in blue, richness prediction from ACE is colored in red, and richness observed is colored in grey. Sample numbers indicating the different management types are given below the graph. A description of the samples is shown in Table 1.

To determine rarefaction curves, richness, and diversity, OTUs were identified at genetic distances of 3, 5, and 20% by using 22,000 randomly selected and denoised sequences per sample. At 20% sequence divergence most rarefaction curves reached saturation, indicating that the surveying effort covered almost the full extent of taxonomic diversity at this genetic distance (Figure S1). Comparison of the rarefaction analyses with the number of OTUs determined by Chao1 and ACE richness estimators revealed that 50.0 to 100% (20% genetic distance) of the estimated taxonomic richness was covered by the surveying effort (Table S3). At 3 and 5% genetic distance, the rarefaction curves were not saturated and the richness estimators indicated that 35.5 to 89.3% and 38.9 to 84.8% of the estimated richness, respectively, were recovered by the sequencing effort (Figures 1, 2 and S1, and Table S3). Thus, we did not survey the full extent of taxonomic diversity at these genetic distances, but a substantial fraction of the bacterial diversity within individual soil samples was assessed at species and genus level by the surveying effort (Figure 1 and Table S3). The comparison of mean Chao1 richness estimates of all forest soils with all grassland soils showed similar values at genetic distances of 3% (3,219 OTUs and 2,611 OTUs, respectively) and 5% (2,331 OTUs and 2,095 OTUs, respectively) but at a genetic distance of 20% (75 OTUs and 153 OTUs, respectively) the richness was higher in grassland (*P*<0.05). The analysis of differences of richness estimates at genetic distances of 3% and 20% and the six management types by employing one-way analysis of variance showed that the analyzed management types did not vary significantly in the predicted number of OTUs (*P*>0.05 in both cases). Comparing this result to previous studies is difficult, as the number of analyzed sequences per sample has an effect on the predicted number of OTUs. In addition, denoising of amplicon sequences was not performed in other studies employing soil-derived pyrosequencing datasets [3], [24]. In our study, richness estimates at 3% sequence divergence were approximately 2-fold higher in non-denoised datasets than in the corresponding denoised datasets (data not shown). In addition, in most other studies far fewer 16S rRNA fragments derived from a few soil samples have been analyzed.

The Shannon index of diversity (H') was determined for all samples (Table S3). At a genetic distance of 3%, the Shannon index ranged from 4.96 to 5.92 in the grassland samples and from 4.74 to 5.99 in the forest samples. Comparison of the mean H' of the different management types revealed that the highest bacterial diversity at a genetic distance of 3% was found in unmanaged beech forest, followed by fertilized intensely managed grassland, fertilized mown pastures grazed by horse and cattle, beech age class forest, spruce age class forest, and unfertilized pastures grazed by sheep (Table S3). In forest soils, the sample with the lowest pH (SAF1; pH 3.3) showed the lowest predicted diversity of all forest samples at all analyzed genetic distances (Figures 1, 2 and S1, and Table S3). Similar results were obtained by Fierer and Jackson [25] but a peak of diversity in soils with near-neutral pH values (BAF1 and BF3) that has been found in other studies [7] was not recorded. The spruce forest samples SAF2 and SAF3 showed higher diversity and richness estimates at phylum level but lower richness estimates at species level than the beech forest samples (Figure 2 and Table S3). Thus, an influence of the tree species on bacterial diversity is indicated. In addition, the rarefaction curves and the H' values derived from beech age class forest soils and unmanaged beech forest soils were not separated at all analyzed genetic distances (Figures 1 and S1, and Table S3), indicating that harvesting type (age class forest or unmanaged forest) has a minor or no impact on overall bacterial diversity and richness.

In grassland soils, similar values for estimated bacterial richness were obtained for the three samples derived from fertilized mown pastures grazed by horse and cattle whereas the replicated samples from the other two management types showed strong variations in estimated bacterial richness (Figure 2 and Table S3). At a genetic distance of 3%, the highest average bacterial richness according to Chao1 richness estimator was predicted for fertilized intensely managed grassland (2,887 OTUs), followed by fertilized mown pastures grazed by horse and cattle (2,720 OTUs), and unfertilized pastures grazed by sheep (2,226 OTUs). Nevertheless, the soil sample UPG3 derived from an unfertilized pasture grazed by sheep showed the second highest OTU estimate of all grassland soils (3,413 OTUs). Thus, bacterial diversity showed strong variations within management types in grassland soils.

### Distribution of taxa and phylotypes across all samples

**Figure 3:**
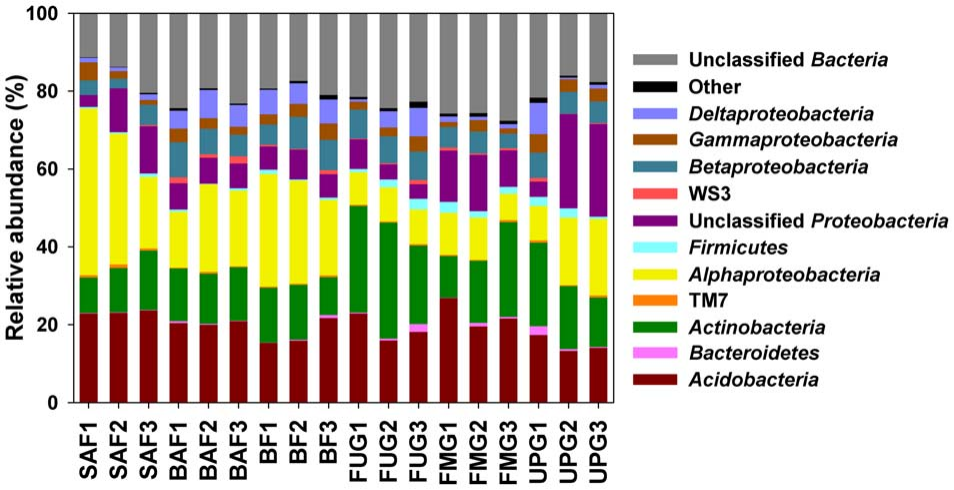
Relative abundances of phylogenetic groups in soils derived from the different grassland and forest sampling sites. Sample numbers indicating the different management types are given below the graph. A description of the samples is shown in Table 1. Phylogenetic groups accounting for ≤0.4% of all classified sequences are summarized in the artificial group ‘others’.

**Figure 4:**
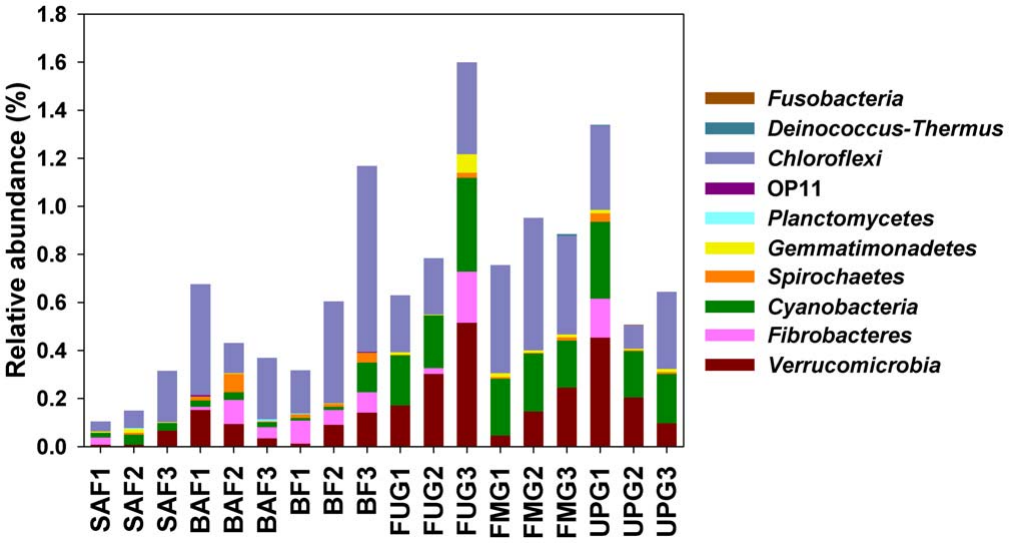
Relative abundances of rare phylogenetic groups of all sequences that were assigned to the domain Bacteria in soils derived from the different grassland and forest sampling sites. A description of the samples is shown in Table 1.

The 474,868 sequences classified below domain level were affiliated to 17 bacterial phyla and 4 proteobacterial classes (Tables S4 and S5). The dominant phyla and proteobacterial classes across all samples were *Acidobacteria*, *Alphaproteobacteria*, *Actinobacteria*, *Betaproteobacteria*, *Deltaproteobacteria*, *Gammaproteobacteria*, and *Firmicutes*, representing 19.6, 18.3, 16.1, 5.9, 3.4, 2.9, and 1.2%, respectively, of all sequences that were assigned to the domain Bacteria. The dominant taxa were present in all samples and corresponded roughly with those reported in other studies on soil bacterial community composition [26]. The members of rare phyla (<1% of all classified sequences) included WS3, *Bacteroidetes*, TM7, *Chloroflexi*, *Verrucomicrobia*, *Cyanobacteria*, *Fibrobacteres*, *Spirochaetes*, *Gemmatimonadetes*, *Planctomyces*, OP11, *Deinococcus*-*Thermus*, and *Fusobacteria* (Figures 3 and 4, and Tables S4 and S5). The most abundant phylotype at a genetic distance of 3% across all samples was an unclassified member of the *Alphaproteobacteria*, representing 2.9% of all sequences. The most abundant phylotype at a genetic distance of 3% within one individual forest soil sample (SAF1) was a member of the family *Caulobacteraceae*, representing 7.9% of the sequences from that soil. In grassland, an unclassified member of the *Proteobacteria* was the predominant phylotype (22.5% of all sequences) within an individual soil sample (UPG2).

### Differences in community structure between forest and grassland soils

**Figure 5:**
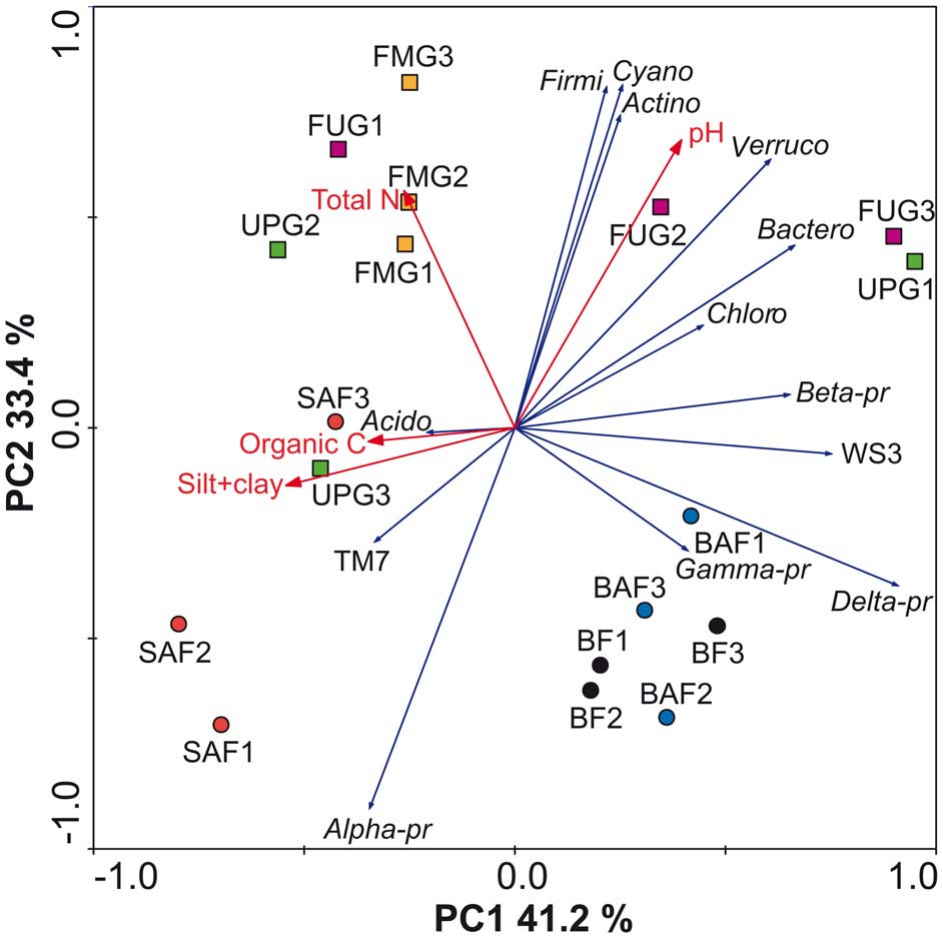
Principal components analysis of bacterial communities as affected by land use, based on the relative abundance of bacterial phyla and proteobacterial classes. Every vector points to the direction of increase for a given variable so that soil samples with similar bacterial communities are localized in similar positions in the diagram. The spruce age class forest (SAF1-3), beech age class forest (BAF1-3), and unmanaged beech forest (BF1-3) sampling sites are marked by the red, green, and black circles, respectively. The fertilized intensely managed grassland (FUG1-3), fertilized mown pasture grazed by horse and cattle (FMG1-3), and unfertilized pasture grazed by sheep (UPG1-3) sampling sites are depicted by red, green, and black squares, respectively. Abbreviations in figure: *Firmi*, *Firmicutes*; *Cyano*, *Cyanobacteria*; *Actino*, *Actinobacteria*; *Verruco*, *Verrucomicrobia*; *Bactero*, *Bacteroidetes*; *Chloro*, *Chloroflexi*; *Beta-pr*, *Betaproteobacteria*; *Delta-pr*, *Deltaproteobacteria*; *Gamma-pr*, *Gammaproteobacteria*; *Alpha-pr*, *Alphaproteobacteria*; *Acido*, *Acidobacteria*.

The relative abundances of dominant taxa varied between grassland and forest soils. The dominant taxa in forest soils were *Alphaproteobacteria* (25.1±8.9%), *Acidobacteria* (20.4±3.0%), *Actinobacteria* (12.7±2.1%), and *Betaproteobacteria* (6.0±2.1%), whereas in grassland soils the predominant phylogenetic group was *Actinobacteria* (19.6±6.5%) followed by *Acidobacteria* (18.7±4.4%), *Alphaproteobacteria* (11.4±4.4%), and *Betaproteobacteria* (5.9%±1.2) (Figure 3, and Tables S4 and S5). The bacterial phyla and proteobacterial classes observed in our forest and grassland soils were also present in similar relative abundances in a meta-analysis of 32 bacterial 16S rRNA gene libraries derived from a variety of different soils, including samples from pristine forest, grassland and agricultural soils [26]. Principal components analysis (PCA) based on the relative abundances of the different bacterial phyla and proteobacterial classes confirmed that the bacterial communities in grassland soils, except the one in sample UPG3, differed from communities in forest soils (Figure 5). We observed significant higher relative abundances of *Actinobacteria*, *Firmicutes*, *Verrucomicrobia*, *Cyanobacteria*, and *Gemmatimonadetes* in grassland soils than in forest soils whereas *Alphaproteobacteria* showed the opposite pattern (*P*<0.05 in all cases) (Figures 3 and 4). Thus, the shifts in soil bacterial community composition correlated with a change from forest to grassland. A similar trend was also found by comparison of Typic Placandept soils derived from a forest site and a pasture grazed by cattle [27]. In addition, sequences affiliated to *Alphaproteobacteria* dominated in 16S rRNA clone libraries of a spruce-fir-beech forest soil in Austria as well as in a Canadian boreal forest soil [14], [28].

Differences of bacterial community structure between grassland and forest soils were also found in the phylogenetic structure within individual lineages. Members of the phylum *Acidobacteria* were predominant across all samples and the second most abundant group in forest and grassland soils, representing approximately 20% of all classified sequences. Correspondingly, members of this phylum have been reported to constitute an average of 20% in bacterial communities derived from various soils [29]. Based on their abundance and the presence in various soil types, *Acidobacteria* appear to play an important role in ecosystem functions of soils, but little is known about physiology and metabolic functions of acidobacterial species. The phylum *Acidobacteria* is divided into 26 subgroups [30] with subgroups 1, 2, 3, 4, and 6 being most abundant within a variety of diverse soils [26], [31]. Here, we detected 18 and 22 of these subgroups in grassland soils and forest soils, respectively. Most abundant in the grasslands soils were subgroups 16, 6, 4, 3, and 7, which represented 6.8, 4.4, 2.8, 1.8, and 1.4%, respectively, of all sequences that were classified in grassland. In forest soils, the dominant subgroups were 3, 16, 6, 1, and 4, representing 7.0, 3.0, 2.9, 2.9, and 2.1%, respectively, of all sequences that were classified (Tables S6 and S7).

Most of the sequences belonging to the second most abundant phylum *Alphaproteobacteria* across all samples were affiliated on the order level to the *Rhodospirillales* in forest soils and to *Rhizobiales* in grassland soils. *Actinobacteridae* and *Rubrobacteridae* were the most abundant subclasses within the *Actinobacteria* in both land use types, but the actinobacterial subclass *Coriobacteridae* was only detected in grassland (Tables S8 and S9). Taking into account that members of this subclass are frequently found in gut or rumen samples [32], [33] it is possible that they were introduced in the grassland sites by cattle or sheep.

**Figure 6:**
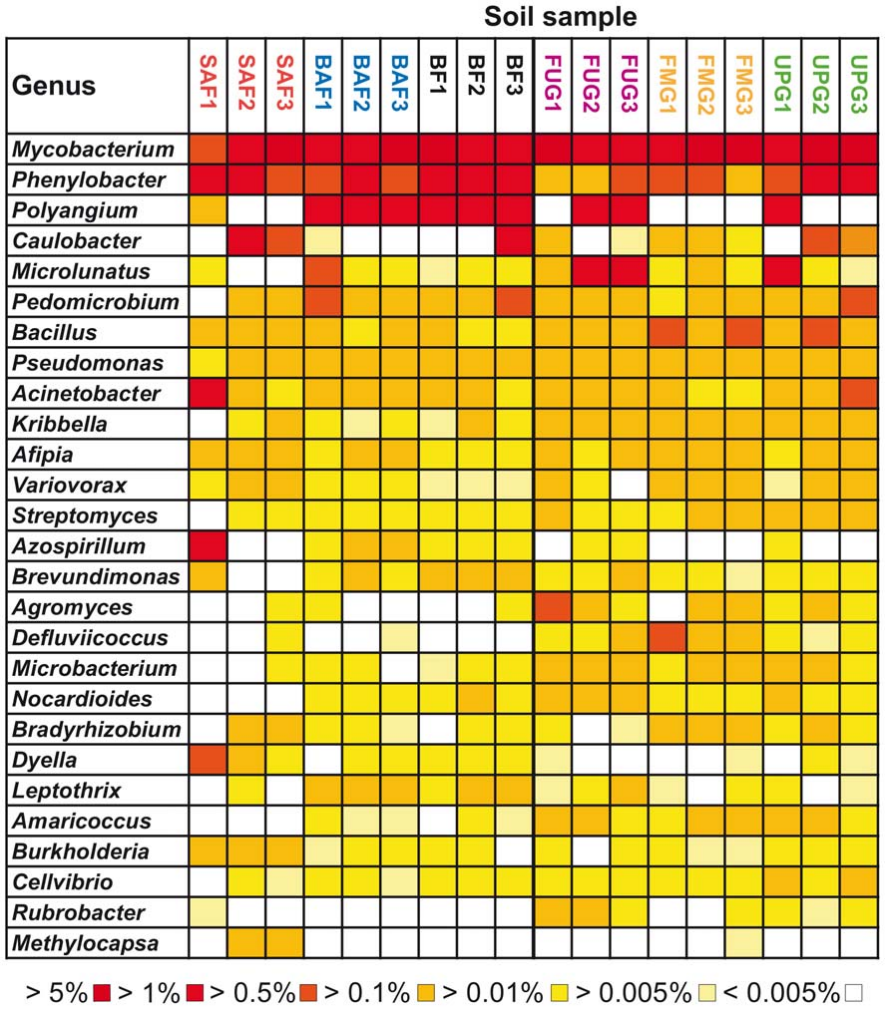
Relative abundances of the most abundant genera as affected by land use. Percentages below the map indicate the abundance of each genus relative to all bacterial sequences that were classified in each of the 18 soils. A description of the samples is shown in Table 1. Grassland and forest samples are separated by a bold line. Samples of different management types are colored in red (SAF1-3), blue (BAF1-3), black (BF1-3), purple (FUG1-3), orange (FMG1-3), and green (UPG1-3).

At the genus level, comparison of the relative abundances revealed significant differences between grassland and forest soil bacterial communities. *Mycobacterium* was the most abundant genus across all soil samples, representing 3.7% of all classified sequences in forest soils and 5.7% in grassland soils. *Mycobacteria* are free-living saprophytes and well adapted to a variety of different environments including soils [34]. The distribution of the other dominant genera *Phenylobacter*, *Bacillus*, *Kribbella*, *Agromyces*, and *Defluviicoccus* varied significantly between forest and grassland soils (*P*<0.05). *Phenylobacter* showed a higher relative abundance in forest soils than in grassland soils whereas *Bacillus*, *Kribbella*, *Agromyces*, and *Defluviicoccus* showed the opposite pattern (Figure 6). *Rubrobacter* and *Streptomyces* were present in higher proportions in grassland soils compared to forest soils (*P*<0.05) (Figure 6). Consistently, Acosta-Martínez et al. [4] found *Rubrobacter* and *Streptomyces* among the top 20 predominant bacteria in two non-disturbed grass systems derived from Texas High Plains.

In summary, significant differences of the community structure between the two analyzed land use types forest and grassland were visible. Here, the different analyzed management types in grassland and forest were not reflected by significant changes in bacterial community structure. Thus, soils derived from an identical management type, i.e., UPG1 to UPG3 do not necessarily harbor similar bacterial communities. An exception was the significant impact of tree species (beech or spruce) on community structure in our forest soils. The comparison of relative abundances of bacterial phyla and proteobacterial classes with respect to tree species revealed significant differences between soils derived from spruce and beech forests (Figure 5). Based upon two sample t-test analyses, *Deltaproteobacteria* were less abundant in spruce forest than in beech forests (*P*<0.05) (Figure 3). At the genus level, *Methylocapsa* and *Burkholderia* were more abundant in spruce forest soil than in beech forest soil, whereas *Nocardioides*, *Leptothrix*, and *Amaricoccus* showed the opposite pattern (Figure 6). Thus, tree species appear to be an important driver of soil bacterial community structure, but the type of harvesting (age class forest or unmanaged forest) does not significantly affect bacterial community composition (Figure 5).

### Impact of soil properties on the relative abundances of bacterial taxa

Previous studies indicated that soil properties such as pH value or soil texture are important drivers of bacterial community structure [12], [35]. We used correlation analysis to identify relationships between the relative abundances of bacterial groups and soil properties. The relative abundances of bacterial groups at different taxonomic levels responded strongly to soil pH. This is in accordance to other surveys on soil bacterial communities derived from different management types in which pH-dependent changes in abundance and distribution of bacterial phyla were observed [36], [37]. At the phylum level, relative abundances of *Bacteroidetes* and *Actinobacteria* in the analyzed soils significantly increased with higher pH values (*P*<0.05 in both cases) (Table 2).

**Table 2:** Spearman's rank correlations between the relative abundances of the six most abundant bacterial phyla and proteobacterial classes and the soil properties in grassland and forest soils.

Taxonomic group	Correlation			
	pH	OC	Total N	Sand/Silt/Clay
*Actinobacteria*	**0.58**	0.26	**0.52**	0.02/−0.08/−0.02
*Bacteroidetes*	**0.71**	0.14	0.33	−0.08/0.17/−0.19
*Alphaproteobacteria*	**−0.68**	0.05	−0.44	−0.12/−0.13/0.22
*Betaproteobacteria*	**0.56**	0.22	0.35	0.04/0.04/0.00
*Deltaproteobacteria*	−0.10	**−0.48**	−0.55	0.43/−0.15/−0.04
*Gammaproteobacteria*	0.27	−0.04	−0.17	−0.13/0.19/−0.19

Correlations for *Acidobacteria* are shown at higher taxonomic resolution Table S10.

Bold numbers: *P*<0.05; Bold and underlined numbers *P*<0.001.

**Figure 7:**
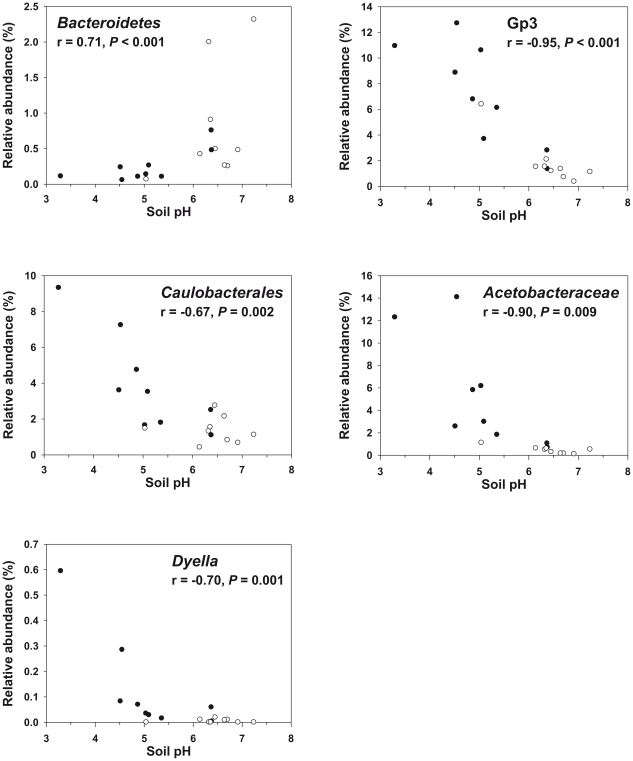
Correlations between relative abundances of different taxonomic groups and soil pH. Black circles represent forest sites and white circles represent grassland sites. Spearman's rank correlation coefficients (r) with the associated *P* values are shown for each taxonomic group. Abbreviation: Gp3, acidobacterial subgroup 3.

As described for a freshwater lake [38] and diverse soils [9], we also found strong correlations of pH and relative abundances of bacterial groups below the phylum level. The relative abundances of the proteobacterial classes *Alphaproteobacteria* and *Betaproteobacteria* were significantly correlated to pH (*P*<0.05). The abundances of *Alphaproteobactia* were negatively correlated with soil pH, whereas the abundances of *Betaproteobacteria* increased with pH (Table 2). Within the *Alphaproteobacteria,* the relative abundances of the order *Caulobacterales* and the family *Acetobacteraceae* showed similar correlations to soil pH as the *Alphaproteobacteria* in general (*P*<0.05 in both cases) (Figure 7). This result corresponded to a cultivation-dependent study of Jimenez-Salgado et al. [39], in which more members of the *Acetobacteraceae* were isolated from low pH soils than from high pH soils. Although relative abundances of *Gammaproteobacteria* showed no significant correlation to soil pH at the class level, the relative abundances of the gammaproteobacterial genus *Dyella* significantly increased with lower pH values (*P*<0.05) (Figure 7). The genus *Dyella* has been recently described by Xie and Yokota [40]. So far, it includes seven species isolated from soil, but no growth of these isolates below pH 4.0 was described [41], [42]. In contrast, the highest relative abundances for sequences affiliated to the genus *Dyella* (0.6% of all classified sequences) were found in sample SAF1, which exhibited the lowest pH value of all samples (pH 3.3). Furthermore, we obtained the highest relative abundances for genera *Azospirillum* and *Acinetobacter* (each representing more than 0.5% of all classified *Bacteria*) in soil sample SAF1 (Figure 6). Thus, our results might help to identify conditions that are best suited for a targeted cultivation of members belonging to these genera.

The occurrence of several subgroups of the *Acidobacteria*, which were predominant across all samples, was also dependent on soil pH. The relative abundances of acidobacterial subgroups 1, 3, 6, 13, 17, and 18 showed strong significant correlations to soil pH (*P*<0.001 in all cases). The relative abundances of subgroups 1, 3, and 13 decreased with pH whereas those of subgroups 6, 17, and 18 were positively correlated with pH (Figure 7 and Table S10). Similar correlations of soil pH and the abundances of acidobacterial subgroups 1, 3, 6, 13, 17, and 18 have been reported by Jones et al. [9]. In addition, the inverse relationship of soil pH on the abundance of members affiliated to subgroup 1 has been reported for soils derived from rotationally grazed perennial ryegrass and white clover pasture [43].

In general, more groups at different taxonomic levels showed significant correlations to soil pH in forest soils than in grassland soils (data not shown). This might be due to the different pH range covered by the analyzed forest and grassland soils. The pH in our forest samples ranged from pH 3.30 to 6.37 (Table 1) whereas the pH values of the grassland samples were all, except sample FMG1, near neutral. Thus, a relatively small pH range was covered by our grassland samples (Table 1), so there is simply less pH range from which to determine correlations. Significant correlations of relative abundances with other soil properties were found for *Deltaproteobacteria* and *Actinobacteria*. The *Deltaproteobacteria* showed a significant correlation to OC (*P*<0.05) with higher abundances in soils with low OC content, whereas *Actinobacteria* showed a significant correlation to total N (*P*<0.05) with higher abundances in soils with high total N content (Table 2), but a connection to the observed correlations was not evident.

### Conclusion

The analysis of one of the largest bacterial 16S rRNA-based datasets from soils revealed statistically significant differences in soil bacterial diversity and community structure between the two land use types forest and grassland. Additionally, the occurrence of different tree species had statistically significant effects on soil bacterial diversity, richness, and community composition in forest. The analysis of influences of soil properties on bacterial community structure revealed that pH had the strongest effect on bacterial community structure of the analyzed soil properties. Management type and other soil properties appear to have a minor impact on soil bacterial community structure and diversity.

In this survey, the correlations between land use type and community composition were obvious. The relative abundances of a number of taxonomic groups changed significantly between forest and grassland soils (e.g., *Actinobacteria*), but the abundances of other taxa (e.g., *Gammaproteobacteria*) were almost unaffected by land use type, indicating that the abundances of the latter groups are influenced by other factors. Specific bacterial groups such as *Amaricoccus* or *Methylocapsa* showed significantly higher abundances in beech or spruce forest soils. Finally, we cannot determine whether pH has a direct or indirect effect on community composition, as a number of soil properties (e.g., OC) are directly or indirectly related to pH [44]. Thus, the effect of a number of different factors is reflected by soil pH and these factors may also drive community composition.

### Availability

The 18 pyrosequencing-derived 16S rRNA gene sequence datasets have been deposited in the GenBank short-read archive under accession number SRA022075.

## Materials and Methods

### Site description, sampling, DNA extraction, and soil characterization

In the frame of the German Biodiversity Exploratories, initiative soil samples were collected from 9 forest and 9 grassland plots of the German Biodiversity Exploratory Schwäbische Alb. The Schwäbische Alb covers more than 450 km×450 km in the state of Baden-Württemberg (southwestern Germany). Soil samples were collected in April 2008. The forest sampling sites included 3 spruce age class forests (SAF1-3), 3 beech age class forests (BAF1-3), and 3 unmanaged beech forests (BF1-3). Grassland sampling sites comprised 3 fertilized intensely managed grasslands (FUG1-3), 3 fertilized mown pastures grazed by horse and cattle (FMG1-3), and 3 unfertilized pastures grazed by sheep (UPG1-3) (Table S1). The dominant grasses included *Poa trivialis*, *Trisetum flavescens*, and *Arrhenaterum elatius* in sites FUG1-3, *Poa trivialis*, *Alopecurus pratensis*, *Trisetum flavescens*, *Dactylis glomerata*, *Festuca pratensis*, *Lolium perenne*, and *Arrhenaterum elatius* in sites FMG1-3, and *Brachypodium pinnatum*, *Bromus erectus*, and *Festuca guestfalica* in sites UPG1-3. A detailed description of the dominant grasses of the individual plots is provided in Table S11.

Soil samples were collected and classified at each of the grassland and forest sites as described by Will et al. [45]. Briefly, five soil cores (8.3 cm in diameter) were sampled with a motor driven soil column cylinder at each corner and in the center of each plot within a given area of 20 m×20 m. Composite samples of the five collected A horizons per plot were used for DNA extraction, after the soils were homogenized and coarse roots and stones (>5 mm) were removed. Total microbial community DNA was extracted from approximately 8 g soil derived from the A horizons of each plot by employing the MoBio PowerMax Soil DNA isolation kit (MoBio Laboratories, Carlsbad, CA, USA) as recommended by the manufacturer. DNA concentrations were quantified by using a NanoDrop ND-1000 UV-Vis Spectrophotometer (NanoDrop Technologies, USA) according to the manufacturer's protocol.

OC content, total N content, soil texture, and soil pH were measured as described by Will et al. [45]. To determine the gravimetric water content, 10 g of moist soil were dried to constant weight at 105°C for 24 h. The mass of water was calculated per mass of dry soil.

### Amplification of 16S rRNA genes and pyrosequencing

The V2-V3 region of the 16S rRNA gene was amplified by PCR. The PCR reaction mixture (33 µl) contained 3.3 µl 10-fold reaction buffer (Fusion GC buffer, FINNZYMES, Espoo, Finland), 800 µM of each of the four deoxynucleoside triphosphates, 3% DMSO, 1.2 µM of each of the primers, 0.5 U of Phusion hot start high-fidelity DNA Polymerase (FINNZYMES), and 20 ng of isolated DNA as template. The V2-V3 region was amplified with the following set of primers containing the Roche 454 pyrosequencing adaptors (underlined): V2for 5′-GCCTCCCTCGCGCCATCAGAGTGGCGGACGGGTGAGTAA-3′ and V3rev 5′-GCCTTGCCAGCCCGCTCAGCGTATTACCGCGGCTGCTG-3′ (modified from Schmalenberger et al. [46]). The following thermal cycling scheme was used: initial denaturation at 98°C for 5 min, 25 cycles of denaturation at 98°C for 45 s, annealing at 68°C for 45 s, and extension at 72°C for 25 s followed by a final extension period at 72°C for 5 min. All samples were amplified in triplicate, pooled in equal amounts, and purified using the peqGold gel extraction kit as recommended by the manufacturer (Peqlab Biotechnologie GmbH, Erlangen, Germany). Quantification of the PCR products was performed using the Quant-iT dsDNA BR assay kit and a Qubit fluorometer (Invitrogen GmbH, Karlsruhe, Germany) as recommended by the manufacturer. The Göttingen Genomics Laboratory determined the sequences of the partial 16S rRNA genes by using a Roche GS-FLX 454 pyrosequencer (Roche, Mannheim, Germany) and the instructions of the manufacturer for amplicon sequencing.

### Analysis of pyrosequencing data

Sequences that were shorter than 200 bp in length and reads containing any unresolved nucleotides were removed from the 18 pyrosequencing-derived datasets. For taxonomy-based analysis, the RDP Classifier of the Ribosomal Database Project (RDP) was used [47] at a confidence threshold of 80%. Pyrosequencing noise was removed for alpha diversity analyses by using the denoiser program [23]. For the determination of OTUs, we defined species, genus, and phylum level at 3, 5, and 20%, respectively, sequence divergence according to Schloss and Handelsman [48]. OTUs were determined for each denoised sequence dataset by using the uclust OTU picker version 1.2.21q of the QIIME software pipeline [49]. We calculated rarefaction curves as well as the Shannon [50] index based on OTU picker data, by employing the RDP pyrosequencing pipeline [51]. ACE and Chao1 [52] indices were calculated using the EstimateS program version 8.2.0 (http://purl.oclc.org/estimates).

### Statistical analyses

Normality tests (Shapiro-Wilk) were performed with data that were used for principal component analysis (PCA), and one-way analysis of variance. Data that did not pass normality test were log transformed and normality test was repeated. Only data that passed normality test were used for further analyses. For each soil attribute and each richness estimate at 3 and 20% genetic distance, one-way analysis of variance and Tukey pair-wise comparisons were used to determine the minimum significant difference (*P*<0.05) between management types by employing STATISTICA 8.0 (StatSoft, Inc., Tulsa, USA). To compare bacterial community structures across all samples based on the relative abundance of bacterial phyla and proteobacterial classes, PCA was performed by using CANOCO for Windows [53]. To correlate bacterial taxonomic groups with soil properties, Spearman's rank correlations were determined by using the SigmaPlot program version 11.0 (Systat Software, Inc., San Jose, CA). We used two sample t-test analyses and M-W-U-Test for non-parametric data to compare relative abundances of bacterial groups and richness estimates between grassland and forest, and on a second level between different management types using the software package PAST [54].

## Supporting Information

Figure S1 Rarefaction curves indicating the observed number of OTUs at genetic distances of 5 and 20% in the different forest and grassland soils. The spruce age class forest (SAF1-3), beech age class forest (BAF1-3), and unmanaged beech forest (BF1-3) sampling sites are marked by the red, blue, and black color, respectively. The fertilized intensely managed grassland (FUG1-3), fertilized mown pasture grazed by horse and cattle (FMG1-3), and unfertilized pasture grazed by sheep (UPG1-3) sampling sites are shown in purple, orange, and green, respectively.
(DOC)

Table S1 Localization of the sampling sites and number of 16S rRNA gene sequences derived from the analyzed grassland and forest soil samples.
(DOC)

Table S2 Mean values of soil properties and standard deviation for each management type and ANOVA *P* values. Differences of soil properties between management types were analyzed by employing one-way analysis of variance and Tukey pair-wise comparisons. Significant ANOVA *P* values are shown in bold (*P*<0.05). Figures followed by different letters indicate differences among management types (*P*<0.05). Abbreviations: SAF, spruce age class forest; BAF, beech age class forest; BF, unmanaged beech forest; FUG, fertilized intensely managed grassland; FMG, fertilized mown pasture grazed by horse and cattle; UPG, unfertilized pasture grazed by sheep. Complete soil and site information for all 18 sampling sites is provided in Table 1.
(DOC)

Table S3 Bacterial diversity as assessed by Shannon index (H') and species richness estimation in all forest and grassland soils. The results from the rarefaction analyses are also depicted in Figure 1 and Figure S1.
(DOC)

Table S4 Relative abundances of bacterial phyla and proteobacterial classes in the analyzed forest soils. Values represent percentages of all sequences assigned to the domain Bacteria for all forest soils or individual forest soils. Groups labeled with asterisks could not be assigned to a specific phylum or a proteobacterial class.
(DOC)

Table S5 Relative abundances of bacterial phyla and proteobacterial classes in the analyzed grassland soils. Values represent percentages of all sequences assigned to the domain Bacteria for all grassland soils or individual grassland soils. Groups labeled with asterisks could not be assigned to a specific phylum or a proteobacterial class.
(DOC)

Table S6 Relative abundances of acidobacterial subgroups in the analyzed forest soils. Values represent percentages of all sequences assigned to the domain Bacteria for all forest soils or individual forest soils. Groups labeled with asterisks could be assigned to the phylum level only.
(DOC)

Table S7 Relative abundances of acidobacterial subgroups in the analyzed grassland soils. Values represent percentages of all sequences assigned to the domain Bacteria for all grassland soils or individual grassland soils. Groups labeled with asterisks could be assigned to the phylum level only.
(DOC)

Table S8 Relative abundances of taxonomic groups within the phylum *Actinobacteria* and within proteobacterial classes in the analyzed forest soils. Values represent percentages of all sequences assigned to the domain Bacteria for all forest soils or individual forest soils. Groups labeled with asterisks could be assigned to the phylum level only.
(DOC)

Table S9 Relative abundances of taxonomic groups within the phylum *Actinobacteria* and within proteobacterial classes in the analyzed grassland soils. Values represent percentages of all sequences assigned to the domain Bacteria for all grassland soils or individual grassland soils. Groups labeled with asterisks could be assigned to the phylum level only.
(DOC)

Table S10 Spearman's rank correlations between relative abundances of *Acidobacteria* subgroups and soil properties. Only relative abundances of acidobacterial subgroups that represented ≥0.029% of all analyzed sequences were considered.
(DOC)

Table S11 Dominant grasses of the analyzed grassland sites.
(DOC)
