# Supplementary material for: Pyrosequencing-Based Assessment of Bacterial Community Structure Along Different Management Types in German Forest and Grassland Soils
Source: PLoS One. 2011 Feb 16;6(2):e17000. doi: 10.1371/journal.pone.0017000 (PMC3040199; doi:10.1371/journal.pone.0017000)
Supplement: Figure S1 — Rarefaction curves indicating the observed number of OTUs at genetic distances of 5 and 20% in the different forest and grassland soils. The spruce age class forest (SAF1-3), beech age class forest (BAF1-3), and unmanaged beech forest (BF1-3) sampling sites are marked by the red, blue, and black color, respectively. The fertilized intensely managed grassland (FUG1-3), fertilized mown pasture grazed by horse and cattle (FMG1-3), and unfertilized pasture grazed by sheep (UPG1-3) sampling sites are shown in purple, orange, and green, respectively. (DOC) [file pone.0017000.s001.doc]

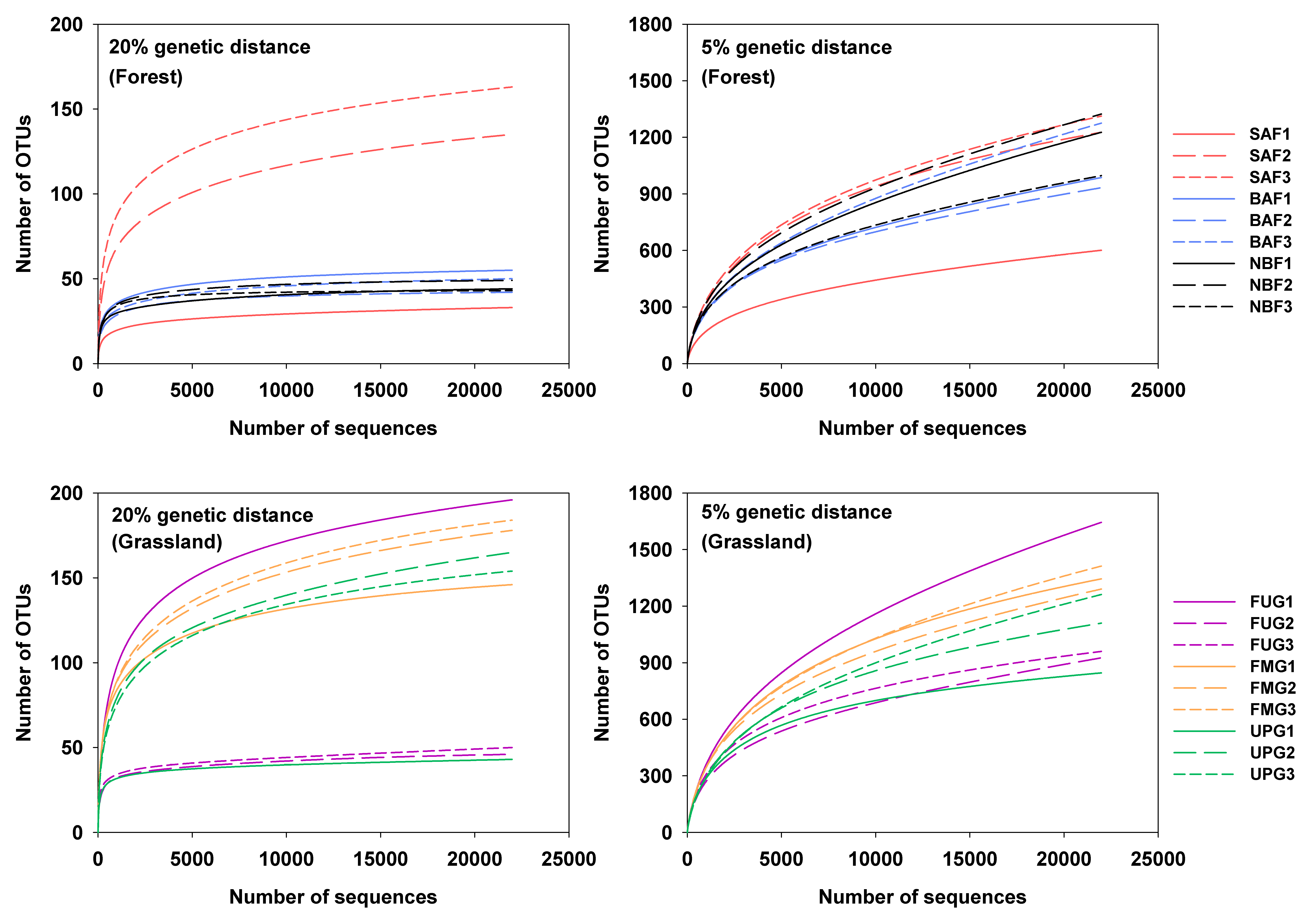


**Figure S1. Rarefaction curves indicating the observed number of OTUs at genetic distances of 5 and 20% in different forest and grassland soils.** The spruce age class forest (SAF1-3), beech age class forest (BAF1-3), and unmanaged beech forest (BF1-3) sampling sites are marked by the red, blue, and black color, respectively. The fertilized intensely managed grassland (FUG1-3), fertilized mown pasture grazed by horse and cattle (FMG1-3), and unfertilized pasture grazed by sheep (UPG1-3) sampling sites are shown in purple, orange, and green, respectively.
