# Supplementary material for: Pyrosequencing-Based Assessment of Bacterial Community Structure Along Different Management Types in German Forest and Grassland Soils
Source: PLoS One. 2011 Feb 16;6(2):e17000. doi: 10.1371/journal.pone.0017000 (PMC3040199; doi:10.1371/journal.pone.0017000)
Supplement: Table S1 — Localization of the sampling sites and number of 16S rRNA gene sequences derived from the analyzed grassland and forest soil samples. (DOC) [file pone.0017000.s002.doc]

**Table S1.** Localization of the sampling sites and number of 16S rRNA gene sequences derived from the analyzed grassland and forest soil samples.

| **Management type** | **Sample** | **Localization of the plots (deg min sec)** | | **No. of sequences ≥ 200 bp** | **No. of sequences assigned to domain Bacteria** | **No. of sequences classified below domain level** |
| --- | --- | --- | --- | --- | --- | --- |
|  |  | **Latitude (N)** | **Longitude (E)** |  |  |  |
| Spruce age class forest | SAF1 | 48 28 41.0628 | 9 20 3.876 | 37,861 | 37,861 | 33,562 |
| Spruce age class forest | SAF2 | 48 22 47.9634 | 9 21 5.2236 | 25,987 | 25,974 | 22,399 |
| Spruce age class forest | SAF3 | 48 24 44.1468 | 9 21 20.1276 | 34,903 | 34,812 | 27,681 |
| Beech age class forest | BAF1 | 48 23 56.7558 | 9 14 41.3772 | 31,373 | 31,372 | 23,723 |
| Beech age class forest | BAF2 | 48 25 10.6242 | 9 24 52.8552 | 34,095 | 34,092 | 27,518 |
| Beech age class forest | BAF3 | 48 23 38.583 | 9 26 45.3732 | 38,580 | 38,580 | 29,621 |
| Unmanaged beech forest | BF1 | 48 23 46.4562 | 9 15 40.881 | 33,298 | 33,298 | 26,859 |
| Unmanaged beech forest | BF2 | 48 22 57.324 | 9 22 56.5824 | 35,373 | 35,372 | 29,228 |
| Unmanaged beech forest | BF3 | 48 22 9.6456 | 9 24 54.7842 | 33,974 | 33,974 | 26,839 |
| Fertilized intensely managed grassland | FUG1 | 48 23 52.8174 | 9 20 31.1526 | 29,890 | 29,865 | 23,444 |
| Fertilized intensely managed grassland | FUG2 | 48 22 36.6852 | 9 28 22.0224 | 31,483 | 31,481 | 23,806 |
| Fertilized intensely managed grassland | FUG3 | 48 24 31.9716 | 9 31 56.5644 | 23,519 | 23,515 | 18,173 |
| Fertilized mown pasture, horse and cattle | FMG1 | 48 22 51.1962 | 9 25 8.0004 | 30,498 | 30,471 | 22,617 |
| Fertilized mown pasture, horse and cattle | FMG2 | 48 23 45.1536 | 9 26 21.1236 | 37,798 | 37,745 | 28,060 |
| Fertilized mown pasture, horse and cattle | FMG3 | 48 24 4.5432 | 9 26 30.0402 | 39,273 | 39,259 | 28,413 |
| Unfertilized pasture, sheep | UPG1 | 48 23 29.1156 | 9 22 36.6486 | 26,510 | 26,500 | 20,761 |
| Unfertilized pasture, sheep | UPG2 | 48 25 21.504 | 9 29 31.6494 | 36,854 | 36,790 | 30,888 |
| Unfertilized pasture, sheep | UPG3 | 48 23 40.8156 | 9 30 10.0506 | 38,015 | 38,001 | 31,276 |
