# Supplementary material for: Pyrosequencing-Based Assessment of Bacterial Community Structure Along Different Management Types in German Forest and Grassland Soils
Source: PLoS One. 2011 Feb 16;6(2):e17000. doi: 10.1371/journal.pone.0017000 (PMC3040199; doi:10.1371/journal.pone.0017000)
Supplement: Table S2 — Mean values of soil properties and standard deviation for each management type and ANOVA P values. Differences of soil properties between management types were analyzed by employing one-way analysis of variance and Tukey pair-wise comparisons. Significant ANOVA P values are shown in bold (P<0.05). Figures followed by different letters indicate differences among management types (P<0.05). Abbreviations: SAF, spruce age class forest; BAF, beech age class forest; BF, unmanaged beech forest; FUG, fertilized intensely managed grassland; FMG, fertilized mown pasture grazed by horse and cattle; UPG, unfertilized pasture grazed by sheep. Complete soil and site information for all 18 sampling sites is provided in Table 1. (DOC) [file pone.0017000.s003.doc]

**Table S2.** Mean values of soil properties and standard deviation for each management type and ANOVA *P* values. Differences of soil properties between management types were analyzed by employing one-way analysis of variance and Tukey pair-wise comparisons. Significant ANOVA *P* values are shown in bold (*P* < 0.05). Figures followed by different letters indicate differences among management types (*P* < 0.05). Abbreviations: SAF, spruce age class forest; BAF, beech age class forest; BF, unmanaged beech forest; FUG, fertilized intensely managed grassland; FMG, fertilized mown pasture grazed by horse and cattle; UPG, unfertilized pasture grazed by sheep. Complete soil and site information for all 18 sampling sites is provided in Table 1.

| **Soil property** | **Management type** | | | | | | **ANOVA** |
| --- | --- | --- | --- | --- | --- | --- | --- |
|  | **SAF** | **BAF** | **BF** | **FUG** | **FMG** | **UPG** | ***P*** |
| pH | 4.7±0.9 b | 5.9±0.9 ab | 5.9±0.8 ab | 6.7±0.3 a | 6.1±0.7 ab | 6.9±0.4 a | **0.011** |
| Organig C **(**g kg^-1^) | 68.2±5.7 a | 58.4±19.7 a | 80.9±22.7 a | 67.7±12.3 a | 68.3±16.8 a | 63.6±20.7 a | 0.731 |
| Total N **(**g kg^-1^) | 4.5±0.6 a | 4.5±1.4 a | 5.6±1.1 a | 6.7±1.3 a | 6.6±1.3 a | 5.6±1.9 a | 0.247 |
| Sand **(**g kg^-1^) | 43±17.0 a | 75±30.3 a | 66±38.6 a | 67±62.4 a | 56±24 a | 115±145.5 a | 0.967 |
| Silt **(**g kg^-1^) | 520±128.5 a | 565±27.8 a | 387±100.5 a | 546±98.5 a | 554±121.8 a | 533±150.0 a | 0.436 |
| Clay **(**g kg^-1^) | 437±114.0 a | 361±39.5 a | 546±112.8 a | 387±158.0 a | 390±122.5 a | 353±216.6 a | 0.559 |
