# Supplementary material for: Pyrosequencing-Based Assessment of Bacterial Community Structure Along Different Management Types in German Forest and Grassland Soils
Source: PLoS One. 2011 Feb 16;6(2):e17000. doi: 10.1371/journal.pone.0017000 (PMC3040199; doi:10.1371/journal.pone.0017000)
Supplement: Table S3 — Bacterial diversity as assessed by Shannon index (H') and species richness estimation in all forest and grassland soils. The results from the rarefaction analyses are also depicted in Figure 1 and Figure S1. (DOC) [file pone.0017000.s004.doc]

**Table S3.** Bacterial diversity as assessed by Shannon index (H´) and species richness estimation in all forest and grassland soils. The results from the rarefaction analyses are also depicted in Figure 1 and Figure S1.

| **Management type** | **Sample** | **Genetic distance (%)** | **H´** | **Rarefaction** | **Chao1** | **ACE** |
| --- | --- | --- | --- | --- | --- | --- |
|  |  |  |  | **No. of operational taxonomic units** | | |
| Spruce age class forest | SAF1 | 3 | 4.74 | 810 | 1625 | 1608 |
|  |  | 5 | 4.21 | 601 | 1119 | 1108 |
|  |  | 20 | 1.62 | 33 | 37 | 36 |
| Spruce age class forest | SAF2 | 3 | 5.75 | 1509 | 2214 | 1924 |
|  |  | 5 | 5.40 | 1227 | 1786 | 1781 |
|  |  | 20 | 2.98 | 135 | 149 | 154 |
| Spruce age class forest | SAF3 | 3 | 5.81 | 1584 | 2745 | 2735 |
|  |  | 5 | 5.55 | 1313 | 2137 | 2144 |
|  |  | 20 | 3.66 | 163 | 193 | 183 |
| Beech age class forest | BAF1 | 3 | 5.55 | 1192 | 3112 | 3076 |
|  |  | 5 | 5.21 | 987 | 2328 | 2305 |
|  |  | 20 | 2.50 | 55 | 60 | 58 |
| Beech age class forest | BAF2 | 3 | 5.46 | 1134 | 3103 | 3056 |
|  |  | 5 | 5.13 | 933 | 2399 | 2366 |
|  |  | 20 | 2.06 | 42 | 42 | 43 |
| Beech age class forest | BAF3 | 3 | 5.87 | 1669 | 4703 | 4669 |
|  |  | 5 | 5.37 | 1276 | 3127 | 3107 |
|  |  | 20 | 2.26 | 50 | 53 | 53 |
| Unmanaged beech forest | BF1 | 3 | 5.68 | 1594 | 4050 | 4024 |
|  |  | 5 | 5.23 | 1227 | 2751 | 2735 |
|  |  | 20 | 2.00 | 44 | 45 | 46 |
| Unmanaged beech forest | BF2 | 3 | 5.99 | 1734 | 4056 | 4033 |
|  |  | 5 | 5.52 | 1324 | 3072 | 3054 |
|  |  | 20 | 2.22 | 49 | 49 | 50 |
| Unmanaged beech forest | BF3 | 3 | 5.66 | 1254 | 3366 | 3326 |
|  |  | 5 | 5.24 | 997 | 2263 | 2242 |
|  |  | 20 | 2.33 | 43 | 43 | 43 |

Table S3 (continued)

| **Management type** | **Sample** | **Genetic distance (%)** | **H´** | **Rarefaction** | **Chao1** | **ACE** |
| --- | --- | --- | --- | --- | --- | --- |
|  |  |  |  | **No. of operational taxonomic units** | | |
| Fertilized intensely managed grassland | FUG1 | 3 | 5.86 | 1960 | 4203 | 4188 |
|  |  | 5 | 5.55 | 1645 | 3236 | 3224 |
|  |  | 20 | 3.47 | 196 | 234 | 221 |
| Fertilized intensely managed grassland | FUG2 | 3 | 5.46 | 1134 | 2807 | 2773 |
|  |  | 5 | 5.10 | 926 | 2163 | 2139 |
|  |  | 20 | 2.35 | 46 | 48 | 47 |
| Fertilized intensely managed grassland | FUG3 | 3 | 5.92 | 1301 | 1652 | 1524 |
|  |  | 5 | 5.52 | 960 | 1359 | 1353 |
|  |  | 20 | 2.60 | 50 | 100 | 73 |
| Fertilized mown pasture, horse and cattle | FMG1 | 3 | 5.79 | 1580 | 2199 | 2017 |
|  |  | 5 | 5.54 | 1345 | 1912 | 1907 |
|  |  | 20 | 3.67 | 146 | 156 | 157 |
| Fertilized mown pasture, horse and cattle | FMG2 | 3 | 5.71 | 1498 | 2938 | 2923 |
|  |  | 5 | 5.53 | 1291 | 2320 | 2309 |
|  |  | 20 | 3.60 | 178 | 207 | 205 |
| Fertilized mown pasture, horse and cattle | FMG3 | 3 | 5.60 | 1648 | 3022 | 3011 |
|  |  | 5 | 5.37 | 1413 | 2561 | 2551 |
|  |  | 20 | 3.50 | 184 | 202 | 209 |
| Unfertilized pasture, sheep | UPG1 | 3 | 5.64 | 1078 | 1320 | 1207 |
|  |  | 5 | 5.35 | 846 | 1119 | 998 |
|  |  | 20 | 2.36 | 43 | 56 | 48 |
| Unfertilized pasture, sheep | UPG2 | 3 | 4.96 | 1302 | 1945 | 1644 |
|  |  | 5 | 4.79 | 1110 | 1681 | 1674 |
|  |  | 20 | 3.23 | 165 | 201 | 198 |
| Unfertilized pasture, sheep | UPG3 | 3 | 4.99 | 1482 | 3413 | 3394 |
|  |  | 5 | 4.81 | 1262 | 2507 | 2495 |
|  |  | 20 | 3.10 | 154 | 171 | 171 |
