# Supplementary material for: Pyrosequencing-Based Assessment of Bacterial Community Structure Along Different Management Types in German Forest and Grassland Soils
Source: PLoS One. 2011 Feb 16;6(2):e17000. doi: 10.1371/journal.pone.0017000 (PMC3040199; doi:10.1371/journal.pone.0017000)
Supplement: Table S4 — Relative abundances of bacterial phyla and proteobacterial classes in the analyzed forest soils. Values represent percentages of all sequences assigned to the domain Bacteria for all forest soils or individual forest soils. Groups labeled with asterisks could not be assigned to a specific phylum or a proteobacterial class. (DOC) [file pone.0017000.s005.doc]

**Table S4.** Relative abundances of bacterial phyla and proteobacterial classes in the analyzed forest soils. Values represent percentages of all sequences assigned to the domain Bacteria for all forest soils or individual forest soils. Groups labeled with asterisks could not be assigned to a specific phylum or a proteobacterial class.

| **Phylogenetic group** | **Relative abundance (%)** | | | | | | | | | |
| --- | --- | --- | --- | --- | --- | --- | --- | --- | --- | --- |
|  | **Average** | **Spruce age class forests** | | | **Beech age class forests** | | | **Unmanaged beech forests** | | |
|  |  | **SAF1** | **SAF2** | **SAF3** | **BAF4** | **BAF5** | **BAF6** | **BF7** | **BF8** | **BF9** |
| *Proteobacteria* | 45.539 | 55.355 | 50.204 | 38.366 | 38.152 | 45.609 | 38.955 | 49.018 | 51.040 | 43.477 |
| *Alphaproteobacteria* | 25.072 | 42.759 | 33.603 | 18.272 | 14.338 | 22.480 | 19.453 | 28.957 | 26.515 | 19.388 |
| *Acidobacteria* | 20.391 | 22.852 | 23.081 | 23.661 | 20.404 | 19.981 | 20.910 | 15.325 | 15.868 | 21.725 |
| *Bacteria** | 18.964 | 11.355 | 13.764 | 20.484 | 24.382 | 19.283 | 23.222 | 19.337 | 17.370 | 21.001 |
| *Actinobacteria* | 12.655 | 9.165 | 11.408 | 15.253 | 13.550 | 12.871 | 13.735 | 14.052 | 14.104 | 9.690 |
| *Proteobacteria** | 7.155 | 3.056 | 11.246 | 12.171 | 6.732 | 6.650 | 6.353 | 5.925 | 7.633 | 5.972 |
| *Betaproteobacteria* | 5.991 | 3.735 | 2.476 | 5.191 | 8.992 | 6.482 | 5.534 | 5.220 | 8.187 | 7.738 |
| *Deltaproteobacteria* | 4.356 | 1.138 | 0.970 | 1.454 | 4.568 | 7.257 | 5.568 | 6.271 | 5.335 | 6.125 |
| *Gammaproteobacteria* | 2.966 | 4.667 | 1.910 | 1.278 | 3.522 | 2.740 | 2.048 | 2.646 | 3.370 | 4.253 |
| WS3 | 0.728 | 0.005 | 0.046 | 0.273 | 1.540 | 0.930 | 1.861 | 0.478 | 0.209 | 1.071 |
| *Firmicutes* | 0.562 | 0.409 | 0.354 | 0.942 | 0.583 | 0.170 | 0.591 | 1.078 | 0.271 | 0.636 |
| TM7 | 0.450 | 0.642 | 0.936 | 0.566 | 0.236 | 0.487 | 0.254 | 0.288 | 0.269 | 0.474 |
| *Chloroflexi* | 0.285 | 0.042 | 0.073 | 0.215 | 0.462 | 0.126 | 0.257 | 0.180 | 0.424 | 0.774 |
| *Bacteroidetes* | 0.249 | 0.111 | 0.058 | 0.138 | 0.478 | 0.238 | 0.104 | 0.105 | 0.263 | 0.756 |
| *Verrucomicrobia* | 0.067 | 0.008 | 0.008 | 0.066 | 0.153 | 0.094 | 0.034 | 0.012 | 0.090 | 0.141 |
| *Fibrobacteres* | 0.049 | 0.029 | 0.000 | 0.000 | 0.013 | 0.100 | 0.047 | 0.096 | 0.062 | 0.085 |
| *Cyanobacteria* | 0.035 | 0.021 | 0.042 | 0.032 | 0.026 | 0.032 | 0.021 | 0.012 | 0.014 | 0.124 |
| *Spirochaetes* | 0.019 | 0.000 | 0.008 | 0.000 | 0.016 | 0.076 | 0.003 | 0.015 | 0.011 | 0.041 |
| *Gemmatimonadetes* | 0.003 | 0.005 | 0.015 | 0.003 | 0.000 | 0.003 | 0.000 | 0.000 | 0.003 | 0.000 |
| *Planctomycetes* | 0.002 | 0.000 | 0.004 | 0.000 | 0.000 | 0.000 | 0.008 | 0.003 | 0.000 | 0.000 |
| OP11 | 0.001 | 0.000 | 0.000 | 0.000 | 0.006 | 0.000 | 0.000 | 0.000 | 0.000 | 0.003 |
