# Supplementary material for: Pyrosequencing-Based Assessment of Bacterial Community Structure Along Different Management Types in German Forest and Grassland Soils
Source: PLoS One. 2011 Feb 16;6(2):e17000. doi: 10.1371/journal.pone.0017000 (PMC3040199; doi:10.1371/journal.pone.0017000)
Supplement: Table S5 — Relative abundances of bacterial phyla and proteobacterial classes in the analyzed grassland soils. Values represent percentages of all sequences assigned to the domain Bacteria for all grassland soils or individual grassland soils. Groups labeled with asterisks could not be assigned to a specific phylum or a proteobacterial class. (DOC) [file pone.0017000.s006.doc]

**Table S5.** Relative abundances of bacterial phyla and proteobacterial classes in the analyzed grassland soils. Values represent percentages of all sequences assigned to the domain Bacteria for all grassland soils or individual grassland soils. Groups labeled with asterisks could not be assigned to a specific phylum or a proteobacterial class.

| **Phylogenetic group** | **Relative abundance (%)** | | | | | | | | | |
| --- | --- | --- | --- | --- | --- | --- | --- | --- | --- | --- |
|  | **Average** | **Fertilized intensely managed grassland** | | | **Fertilized mown pasture, horse and cattle** | | | **Unfertilized pasture, sheep** | | |
|  |  | **FUG1** | **FUG2** | **FUG3** | **FMG4** | **FMG5** | **FMG6** | **UPG7** | **UPG8** | **UPG9** |
| *Proteobacteria* | 34.863 | 26.044 | 26.028 | 31.218 | 31.906 | 34.699 | 22.318 | 31.989 | 50.886 | 53.356 |
| *Bacteria** | 22.542 | 21.500 | 24.380 | 22.717 | 25.775 | 25.659 | 27.627 | 21.657 | 16.042 | 17.697 |
| *Actinobacteria* | 19.625 | 27.420 | 29.796 | 20.174 | 10.722 | 15.949 | 24.374 | 21.460 | 16.167 | 12.684 |
| *Acidobacteria* | 18.710 | 22.873 | 15.946 | 18.150 | 26.835 | 19.571 | 21.572 | 17.306 | 13.226 | 14.034 |
| *Proteobacteria** | 12.437 | 7.604 | 3.888 | 3.776 | 13.190 | 14.428 | 9.361 | 3.921 | 24.224 | 23.799 |
| *Alphaproteobacteria* | 11.434 | 8.321 | 8.758 | 8.930 | 10.768 | 10.862 | 6.765 | 8.830 | 17.374 | 19.636 |
| *Betaproteobacteria* | 5.863 | 7.504 | 6.953 | 7.272 | 5.274 | 5.595 | 3.739 | 6.408 | 5.624 | 5.584 |
| *Gammaproteobacteria* | 2.743 | 2.032 | 2.271 | 3.934 | 1.303 | 2.967 | 1.409 | 4.751 | 3.284 | 3.339 |
| *Deltaproteobacteria* | 2.387 | 0.583 | 4.158 | 7.306 | 1.372 | 0.845 | 1.044 | 8.079 | 0.381 | 0.997 |
| *Firmicutes* | 1.845 | 0.837 | 1.992 | 2.760 | 2.822 | 1.674 | 1.752 | 2.366 | 2.324 | 0.603 |
| *Bacteroidetes* | 0.723 | 0.254 | 0.480 | 1.999 | 0.069 | 0.906 | 0.423 | 2.317 | 0.495 | 0.263 |
| WS3 | 0.459 | 0.161 | 0.305 | 1.050 | 0.791 | 0.376 | 0.540 | 0.992 | 0.068 | 0.192 |
| TM7 | 0.371 | 0.281 | 0.289 | 0.332 | 0.325 | 0.215 | 0.515 | 0.577 | 0.277 | 0.526 |
| *Chloroflexi* | 0.338 | 0.238 | 0.235 | 0.383 | 0.450 | 0.551 | 0.410 | 0.351 | 0.098 | 0.321 |
| *Cyanobacteria* | 0.237 | 0.208 | 0.219 | 0.391 | 0.236 | 0.241 | 0.196 | 0.321 | 0.193 | 0.205 |
| *Verrucomicrobia* | 0.226 | 0.171 | 0.302 | 0.515 | 0.046 | 0.146 | 0.245 | 0.453 | 0.204 | 0.097 |
| *Fibrobacteres* | 0.034 | 0.000 | 0.025 | 0.213 | 0.000 | 0.000 | 0.000 | 0.162 | 0.000 | 0.000 |
| *Gemmatimonadetes* | 0.016 | 0.010 | 0.003 | 0.077 | 0.016 | 0.011 | 0.013 | 0.015 | 0.005 | 0.013 |
| *Spirochaetes* | 0.010 | 0.003 | 0.000 | 0.021 | 0.007 | 0.003 | 0.013 | 0.034 | 0.005 | 0.008 |
| *Deinococcus-Thermus* | 0.001 | 0.000 | 0.000 | 0.000 | 0.000 | 0.000 | 0.008 | 0.003 | 0.000 | 0.000 |
| *Fusobacteria* | 0.0003 | 0.000 | 0.000 | 0.000 | 0.000 | 0.000 | 0.000 | 0.000 | 0.003 | 0.000 |
