# Supplementary material for: Pyrosequencing-Based Assessment of Bacterial Community Structure Along Different Management Types in German Forest and Grassland Soils
Source: PLoS One. 2011 Feb 16;6(2):e17000. doi: 10.1371/journal.pone.0017000 (PMC3040199; doi:10.1371/journal.pone.0017000)
Supplement: Table S6 — Relative abundances of acidobacterial subgroups in the analyzed forest soils. Values represent percentages of all sequences assigned to the domain Bacteria for all forest soils or individual forest soils. Groups labeled with asterisks could be assigned to the phylum level only. (DOC) [file pone.0017000.s007.doc]

**Table S6.** Relative abundances of acidobacterial subgroups in the analyzed forest soils. Values represent percentages of all sequences assigned to the domain Bacteria for all forest soils or individual forest soils. Groups labeled with asterisks could be assigned to the phylum level only.

| **Phylogenetic group** | **Relative abundance (%)** | | | | | | | | | |
| --- | --- | --- | --- | --- | --- | --- | --- | --- | --- | --- |
|  | **Average** | **Spruce age class forests** | | | **Beech age class forests** | | | **Unmanaged beech forests** | | |
|  |  | **SAF1** | **SAF2** | **SAF3** | **BAF4** | **BAF5** | **BAF6** | **BF7** | **BF8** | **BF9** |
| *Acidobacteria* | **20.391** | **22.852** | **23.081** | **23.661** | **20.404** | **19.981** | **20.910** | **15.325** | **15.868** | **21.725** |
| Gp3 | 7.024 | 10.937 | 12.705 | 10.606 | 1.348 | 8.861 | 6.115 | 6.784 | 3.684 | 2.796 |
| Gp16 | 2.951 | 0.103 | 2.495 | 4.010 | 5.049 | 2.256 | 3.595 | 2.204 | 3.017 | 4.080 |
| Gp6 | 2.942 | 0.127 | 0.574 | 1.703 | 8.068 | 1.596 | 1.903 | 1.697 | 4.388 | 6.676 |
| Gp1 | 2.931 | 10.626 | 5.532 | 3.143 | 0.064 | 2.443 | 1.063 | 1.535 | 1.179 | 0.439 |
| Gp4 | 2.121 | 0.164 | 0.543 | 2.172 | 2.630 | 1.296 | 4.217 | 1.009 | 1.563 | 5.101 |
| Gp7 | 1.145 | 0.021 | 0.562 | 1.129 | 1.001 | 2.074 | 2.447 | 1.030 | 0.958 | 0.889 |
| *Acidobacteria** | 0.330 | 0.449 | 0.377 | 0.149 | 0.354 | 0.422 | 0.384 | 0.291 | 0.263 | 0.280 |
| Gp17 | 0.289 | 0.008 | 0.096 | 0.293 | 0.771 | 0.170 | 0.280 | 0.126 | 0.300 | 0.580 |
| Gp5 | 0.201 | 0.032 | 0.108 | 0.178 | 0.233 | 0.340 | 0.241 | 0.258 | 0.136 | 0.286 |
| Gp11 | 0.168 | 0.016 | 0.015 | 0.118 | 0.434 | 0.150 | 0.308 | 0.120 | 0.102 | 0.238 |
| Gp22 | 0.160 | 0.005 | 0.012 | 0.092 | 0.360 | 0.138 | 0.228 | 0.150 | 0.195 | 0.253 |
| Gp10 | 0.037 | 0.011 | 0.000 | 0.006 | 0.032 | 0.070 | 0.044 | 0.060 | 0.051 | 0.056 |
| Gp2 | 0.030 | 0.158 | 0.015 | 0.020 | 0.006 | 0.041 | 0.005 | 0.009 | 0.000 | 0.000 |
| Gp13 | 0.026 | 0.114 | 0.012 | 0.011 | 0.000 | 0.041 | 0.013 | 0.024 | 0.008 | 0.000 |
| Gp15 | 0.022 | 0.026 | 0.035 | 0.020 | 0.010 | 0.038 | 0.041 | 0.006 | 0.011 | 0.006 |
| Gp25 | 0.007 | 0.000 | 0.000 | 0.003 | 0.010 | 0.012 | 0.008 | 0.009 | 0.006 | 0.018 |
| Gp18 | 0.006 | 0.000 | 0.000 | 0.009 | 0.019 | 0.000 | 0.000 | 0.006 | 0.006 | 0.015 |
| Gp14 | 0.006 | 0.045 | 0.000 | 0.000 | 0.000 | 0.000 | 0.000 | 0.000 | 0.000 | 0.000 |
| Gp12 | 0.004 | 0.008 | 0.000 | 0.000 | 0.000 | 0.015 | 0.013 | 0.000 | 0.000 | 0.000 |
| Gp20 | 0.003 | 0.003 | 0.000 | 0.000 | 0.010 | 0.000 | 0.003 | 0.003 | 0.003 | 0.009 |
| Gp8 | 0.002 | 0.000 | 0.000 | 0.000 | 0.000 | 0.018 | 0.000 | 0.003 | 0.000 | 0.000 |
| Gp19 | 0.001 | 0.000 | 0.000 | 0.000 | 0.000 | 0.000 | 0.003 | 0.000 | 0.000 | 0.006 |
| Gp9 | 0.001 | 0.000 | 0.000 | 0.000 | 0.006 | 0.000 | 0.000 | 0.000 | 0.000 | 0.000 |
