# Supplementary material for: Pyrosequencing-Based Assessment of Bacterial Community Structure Along Different Management Types in German Forest and Grassland Soils
Source: PLoS One. 2011 Feb 16;6(2):e17000. doi: 10.1371/journal.pone.0017000 (PMC3040199; doi:10.1371/journal.pone.0017000)
Supplement: Table S7 — Relative abundances of acidobacterial subgroups in the analyzed grassland soils. Values represent percentages of all sequences assigned to the domain Bacteria for all grassland soils or individual grassland soils. Groups labeled with asterisks could be assigned to the phylum level only. (DOC) [file pone.0017000.s008.doc]

**Table S7.** Relative abundances of acidobacterial subgroups in the analyzed grassland soils. Values represent percentages of all sequences assigned to the domain Bacteria for all grassland soils or individual grassland soils. Groups labeled with asterisks could be assigned to the phylum level only.

| **Phylogenetic group** | **Relative abundance (%)** | | | | | | | | | |
| --- | --- | --- | --- | --- | --- | --- | --- | --- | --- | --- |
|  | **Average** | **Fertilized intensely managed grassland** | | | **Fertilized mown pasture, horse and cattle** | | | **Unfertilized pasture, sheep** | | |
|  |  | **FUG1** | **FUG2** | **FUG3** | **FMG4** | **FMG5** | **FMG6** | **UPG7** | **UPG8** | **UPG9** |
| *Acidobacteria* | **18.710** | **22.873** | **15.946** | **18.150** | **26.835** | **19.571** | **21.572** | **17.306** | **13.226** | **14.034** |
| Gp16 | 6.798 | 9.925 | 4.380 | 3.321 | 9.137 | 8.197 | 11.816 | 3.925 | 4.629 | 4.147 |
| Gp6 | 4.408 | 8.331 | 7.967 | 5.192 | 1.549 | 3.624 | 3.724 | 5.113 | 2.422 | 3.100 |
| Gp4 | 2.798 | 1.771 | 1.372 | 3.593 | 3.669 | 2.567 | 2.331 | 3.638 | 3.025 | 3.505 |
| Gp3 | 1.795 | 0.717 | 0.365 | 1.535 | 6.390 | 2.090 | 1.516 | 1.117 | 1.188 | 1.358 |
| Gp7 | 1.386 | 1.005 | 0.588 | 2.135 | 3.771 | 1.258 | 0.963 | 1.721 | 0.889 | 0.784 |
| Gp17 | 0.460 | 0.321 | 0.394 | 0.863 | 0.289 | 0.440 | 0.469 | 0.668 | 0.321 | 0.516 |
| Gp11 | 0.304 | 0.321 | 0.337 | 0.315 | 0.269 | 0.400 | 0.252 | 0.238 | 0.307 | 0.287 |
| Gp5 | 0.221 | 0.080 | 0.098 | 0.621 | 0.167 | 0.400 | 0.143 | 0.275 | 0.247 | 0.071 |
| Gp1 | 0.183 | 0.003 | 0.000 | 0.166 | 1.267 | 0.064 | 0.059 | 0.102 | 0.024 | 0.076 |
| Gp22 | 0.160 | 0.167 | 0.108 | 0.162 | 0.062 | 0.368 | 0.176 | 0.275 | 0.060 | 0.066 |
| *Acidobacteria** | 0.122 | 0.177 | 0.254 | 0.140 | 0.167 | 0.053 | 0.076 | 0.136 | 0.043 | 0.100 |
| Gp18 | 0.024 | 0.010 | 0.029 | 0.034 | 0.010 | 0.042 | 0.015 | 0.038 | 0.030 | 0.011 |
| Gp25 | 0.017 | 0.020 | 0.016 | 0.021 | 0.030 | 0.008 | 0.005 | 0.030 | 0.016 | 0.013 |
| Gp20 | 0.016 | 0.010 | 0.019 | 0.000 | 0.026 | 0.048 | 0.010 | 0.008 | 0.019 | 0.000 |
| Gp10 | 0.006 | 0.003 | 0.010 | 0.043 | 0.000 | 0.000 | 0.000 | 0.015 | 0.000 | 0.000 |
| Gp15 | 0.005 | 0.000 | 0.000 | 0.009 | 0.016 | 0.005 | 0.015 | 0.000 | 0.003 | 0.000 |
| Gp13 | 0.003 | 0.003 | 0.006 | 0.000 | 0.010 | 0.005 | 0.003 | 0.004 | 0.000 | 0.000 |
| Gp9 | 0.002 | 0.007 | 0.003 | 0.000 | 0.000 | 0.000 | 0.000 | 0.004 | 0.003 | 0.000 |
| Gp8 | 0.001 | 0.000 | 0.000 | 0.000 | 0.007 | 0.000 | 0.000 | 0.000 | 0.000 | 0.000 |
