# Supplementary material for: Pyrosequencing-Based Assessment of Bacterial Community Structure Along Different Management Types in German Forest and Grassland Soils
Source: PLoS One. 2011 Feb 16;6(2):e17000. doi: 10.1371/journal.pone.0017000 (PMC3040199; doi:10.1371/journal.pone.0017000)
Supplement: Table S10 — Spearman's rank correlations between relative abundances of Acidobacteria subgroups and soil properties. Only relative abundances of acidobacterial subgroups that represented ≥0.029% of all analyzed sequences were considered. (DOC) [file pone.0017000.s011.doc]

**Table S10.** Spearman’s rank correlations between relative abundances of *Acidobacteria* subgroups and soil properties. Only relative abundances of acidobacterial subgroups that represented ≥ 0.029% of all analyzed sequences were considered.

| ***Acidobacteria* subgroup** | **Correlation** | | | |
| --- | --- | --- | --- | --- |
|  | **pH** | **Organic C** | **Total N** | **Sand/Silt/Clay** |
| 1 | **-0.87** | -0.32 | **-0.71** | 0.02/-0.26/0.23 |
| 2 | None | None | None | None |
| 3 | **-0.95** | -0.21 | **-0.56** | -0.07/-0.22/0.29 |
| 4 | **0.49** | -0.40 | -0.11 | 0.20/0.04/-0.11 |
| 5 | 0.01 | -0.16 | -0.12 | 0.16/-0.15/0.12 |
| 6 | **0.80** | 0.24 | **0.47** | 0.08/0.11/-0.12 |
| 7 | -0.07 | -0.40 | -0.19 | 0.35/-0.06/-0.04 |
| 10 | -0.23 | -0.18 | -0.39 | 0.25/-0.13/0.10 |
| 11 | **0.67** | 0.15 | **0.54** | 0.14/0.43/-0.34 |
| 13 | **-0.75** | -0.20 | -0.45 | 0.25/0.02/-0.09 |
| 16 | **0.60** | 0.22 | **0.64** | 0.01/0.18/-0.15 |
| 17 | **0.77** | 0.03 | 0.30 | 0.02/0.14/-0.16 |
| 18 | **0.76** | 0.20 | **0.49** | 0.05/0.12/-0.19 |
| 22 | 0.41 | 0.09 | 0.15 | 0.34/0.15/-0.22 |

Bold numbers: *P* < 0.05; Bold and underlined numbers: *P* < 0.001. None: subgroup 2 was not detected in grassland.
