# Supplementary material for: Pyrosequencing-Based Assessment of Bacterial Community Structure Along Different Management Types in German Forest and Grassland Soils
Source: PLoS One. 2011 Feb 16;6(2):e17000. doi: 10.1371/journal.pone.0017000 (PMC3040199; doi:10.1371/journal.pone.0017000)
Supplement: Table S11 — Dominant grasses of the analyzed grassland sites. (DOC) [file pone.0017000.s012.doc]

**Table S11.** Dominant grasses of the analyzed grassland sites.

| **Management type** | **Sample** | **Dominant grasses** |
| --- | --- | --- |
| Fertilized intensely managed grassland | FUG1 | *Arrhenaterum elatius*, *Trisetum flavescens*, *Poa trivialis* |
| Fertilized intensely managed grassland | FUG2 | *Poa trivialis*, *Trisetum flavescens* |
| Fertilized intensely managed grassland | FUG3 | *Poa trivialis*, *Trisetum flavescens* |
| Fertilized mown pasture, horse and cattle | FMG1 | *Poa trivialis*, *Alopecurus pratensis*, *Festuca pratensis*, *Lolium perenne* |
| Fertilized mown pasture, horse and cattle | FMG2 | *Poa trivialis*, *Trisetum flavescens*, *Alopecurus pratensis*, *Dactylis glomerata* |
| Fertilized mown pasture, horse and cattle | FMG3 | *Dactylis glomerata*, *Arrhenaterum elatius*, *Trisetum flavescens*, *Poa trivialis*, *Alopecurus pratensis* |
| Unfertilized pasture, sheep | UPG1 | *Brachypodium pinnatum*, *Festuca guestfalica* |
| Unfertilized pasture, sheep | UPG2 | *Bromus erectus* |
| Unfertilized pasture, sheep | UPG3 | *Bromus erectus*, *Brachypodium pinnatum* |
